# Cancer Pain Treatment and Management: An Interprofessional Learning Module for Prelicensure Health Professional Students

**DOI:** 10.15766/mep_2374-8265.10953

**Published:** 2020-09-09

**Authors:** Scott M. Fishman, David Copenhaver, Jennifer M. Mongoven, Kathryn Lorenzen, Ellery Schlingmann, Heather M. Young

**Affiliations:** 1 Professor and Fullerton Endowed Chair in Pain Medicine, Department of Anesthesiology and Pain Medicine, School of Medicine, University of California, Davis; Director, Center for Advancing Pain Relief, Betty Irene Moore School of Nursing and School of Medicine, University of California, Davis; 2 Associate Professor and Chief, Department of Anesthesiology and Pain Medicine, Division of Pain Medicine, School of Medicine, University of California, Davis; Associate Director, Center for Advancing Pain Relief, Betty Irene Moore School of Nursing and School of Medicine, University of California, Davis; 3 Associate Director of Operations, Family Caregiving Institute, Betty Irene Moore School of Nursing, University of California, Davis; 4 Associate Director, Center for Advancing Pain Relief, Betty Irene Moore School of Nursing and School of Medicine, University of California, Davis; 5 Research Associate, Center for Advancing Pain Relief, Betty Irene Moore School of Nursing and School of Medicine, University of California, Davis; 6 Dignity Health Dean's Chair for Nursing Leadership, Associate Vice Chancellor for Nursing, Dean, and Professor, Betty Irene Moore School of Nursing, University of California, Davis

**Keywords:** Interprofessional, Interprofessional Relations, Patient-Centered, Cancer Pain Treatment, Pain Management, Online/Distance Education, Case-Based Learning, Flipped Classroom, Opioids

## Abstract

**Introduction:**

The imperative of medicine is to treat suffering and to cure when possible. This learning module has been designed to expand providers' knowledge of how to sustain life, restore health, relieve suffering, and provide comfort for people who are experiencing cancer-induced pain. The module uses cancer pain as the context through which students can learn interprofessional, team-based, and person-centered approaches to delivery of care.

**Methods:**

Using the facilitator's guide, handouts, and other materials developed for this project, the module can be delivered as an in-person training session (approximately 120 minutes) for small groups of learners (teams of eight to 12 students drawn from multiple health care professions or schools). Prelearning materials and postsession activities are included that can enhance the experience.

**Results:**

This module was developed and tested with two pilot programs that were evaluated with focus groups, direct observation, and a postsession survey completed by learners. Data demonstrated high approval of and appreciation for the content and structure of the module by both learners and facilitators.

**Discussion:**

Many learners work with other health care professionals in their clinical experiences but have not had opportunities to effectively work in interprofessional collaborative practice. This interprofessional education activity allows students from disparate health professions to work together to identify patient-centered treatment options through interprofessional collaborative teamwork in a classroom setting.

## Educational Objectives

After completing this cancer pain treatment and management module, learners will be able to:

1. Construct a problem list and treatment plan for initial management of a complex pain case presentation.
2. Use the biopsychosocial model to create an effective patient-centered pain management plan.
3. In the context of an interprofessional team, adjust a plan in response to feedback gained during ongoing assessment of pain, function, and overall systems.
4. Describe the benefits of patient-centered and team-based care.
5. Communicate with other health professionals in a manner that supports a team approach to care.

## Introduction

There are gaps in interprofessional communication in both education and clinical practice. As articulated in the Josiah Macy Jr. Foundation's January 2013 conference recommendations report *Transforming Patient Care: Aligning Interprofessional Education With Clinical Practice Redesign*, “health professions education and health care practice have developed and functioned separately, with little recognition that the two are inextricably linked.”^1^ Nonetheless, the value of interprofessional education (IPE) and interprofessional care in the health sciences has been widely recognized.^2^ Interactive experiences in which participants learn with and from each other are one approach for improving professional practice and care delivery.^3^

In 2011, the Institute of Medicine (since 2015, the National Academy of Medicine) estimated that roughly 100 million American adults suffered from chronic pain, impacting more Americans than diabetes, heart disease, and cancer combined, with annual direct and indirect costs reaching approximately $600 billion.^4^ These estimates do not include the considerable burdens of acute, pediatric, cancer, or end-of-life related pain.

Pain is the most common reason people visit a health care provider, yet pain management is not emphasized in the curricula of most health care professional schools.^5,6^ Therefore, it is critical that clinicians from a variety of fields learn to participate in the optimal care of patients with chronic pain and pain at the end of life. Optimal pain management requires a team-based approach to care and calls upon multiple professions for expertise. These include, but are not limited to, multiple specialties within medicine (e.g., internal medicine, anesthesiology, psychiatry, oncology), nursing, psychology, social work, pharmacy, physical therapy, dentistry, and complementary and alternative medicine.

The complex clinical phenomenon of cancer pain is particularly appropriate for interprofessional learning with a focus on team-based care. Relieving cancer pain and suffering requires both biomedical expertise in diagnosis and therapeutics and thoughtful attention to an individual's well-being, goals, and preferences.^7^ Cancer pain has profound implications for many areas of a person's life, including personal relationships, work productivity, and the ability to effectively address other health problems. A systematic review conducted by Taplin and colleagues indicated that multidisciplinary care teams improve “quality, access and patient-centered outcomes” in cancer care.^8^ Moreover, a biopsychosocial approach to management that includes both pharmacologic and nonpharmacologic modalities is particularly important for those with pain, as depression is a frequently reported comorbid condition.^9^ Given the complexity of factors contributing to cancer pain, relief requires coordinated and collaborative interprofessional expertise.

This IPE project leveraged an established set of pain management core competencies^5^ and core competencies for interprofessional collaborative practice.^10^ From this starting point, the module introduces an innovative approach to interprofessional, team-based training for prelicensure health professional students that has been developed with a broadly inclusive set of participants, a consensus-based model of decision-making, and competency-based outcomes. This module contributes to a robust body of literature^11^ that supports IPE, including a 2013 report from the Institute of Medicine addressing the potential for IPE to improve patient care and health outcomes.^12^ However, the module can be distinguished from related studies as, to our knowledge, it uniquely emphasizes prelicensure IPE through the lens of cancer pain and treatment. This conclusion is based on searching Google Scholar and PubMed databases for any combination of the search terms *interprofessional, IPE, cancer,* or *pain* and finding no other evidence of IPE programs focused on this subject area. Although this IPE module has been tested in prelicensure settings, it is likely useful for postlicensure learners as well.

This module uses a case study approach featuring a patient who was diagnosed with metastatic prostate cancer 17 years ago. The patient (Gerald) has been using a novel chemotherapeutic agent, and his pain is generally well managed, although recently he has been experiencing significant breakthrough pain. The case references Gerald's increased isolation due to side effects of his medication (e.g., constipation), thereby encouraging students to discuss symptom management, depression, social isolation, and the resources available to address these issues when working on an interprofessional team.

## Methods

### Educational Approach and Rationale Used

The purpose of this module was to increase knowledge of cancer pain management and interprofessional practice competencies using case-based interprofessional discussions. The module was developed by an interprofessional group of faculty clinicians, students, and alumni representing medicine, nursing, pharmacy, psychology, and social work. The effectiveness of the cancer pain treatment and management module was tested in two pilot sessions. In the first pilot, nine learners from five health professions included two nurse practitioner or physician assistant students, three pharmacy students, two medical students, and two social work students. It should be noted that students in the nurse practitioner and physician assistant programs at the University of California, Davis (UC Davis) learn alongside each other; therefore, these disciplines were grouped in reporting.

The module lasted 2 hours and 30 minutes. Implementation directions were provided in the facilitator guide (Appendix A), and we assigned the learners to review the Appendix B module (instructions on how to access this zip file are provided in Appendix C), core competencies and learning goals (Appendix D), the case study (Appendix E), and a presentation detailing the return visit of the study patient (Appendix F). Following implementation of the module and a short break, a 45-minute debriefing session was held with learners, facilitators, and observers. The module incorporated direct instruction, as well as inquiry-based and kinesthetic instructional methods. Two other modules were tested simultaneously, and learners from those sessions were included in the debriefing. The session included time for participants to complete surveys to collect information on their experiences and to solicit feedback using the Pain Knowledge and Beliefs Questionnaire^13^ (Appendix G), developed by an interprofessional faculty team at the University of Toronto to assess an interprofessional undergraduate pain curriculum. The program ended with a facilitated discussion with learners covering key learning points.

The interdisciplinary group that developed the initial pilot and the evaluation team reviewed data from the first pilot to improve the module and design a second pilot. One key change was to utilize a flipped classroom model^14,15^ that provided a blended learning environment, with learning content that was addressed inside the classroom as well as through independent study. In this case, learners were required to review an independent learning activity that provided background on the core subject matter (i.e., cancer pain and treatment options and the biopsychosocial model). With this adjustment, the second in-person pilot session included a greater focus on small-group discussions; learning from, with, and about each profession; and team-based learning.

The second pilot was conducted in January 2016. Its goal was to present the case-based educational module to students and to solicit feedback from both students and facilitators in order to (1) determine if program design changes were supported and successful and (2) obtain further feedback that could be integrated into final project recommendations and guidance documents. The module was again piloted with nine learners from five different professions: three nurse practitioner or physician assistant students, two medical students, two pharmacy residents, and two social work students.

### Module Implementation

Cancer pain management served as the context through which students could learn interprofessional, team-based, person-centered care. A detailed facilitator guide was included (Appendix A). The learning activity targeted two nationally recognized competencies: the core competencies in pain management for prelicensure clinical education^5^ and the core competencies for interprofessional practice.^10^ The specific competencies and learning goals were outlined in Handout I (Appendix D). A variety of instructional methods were used, including independent learning, case-based learning, facilitated small-group discussions, and team-based learning (see Table 1).

**Table 1.**
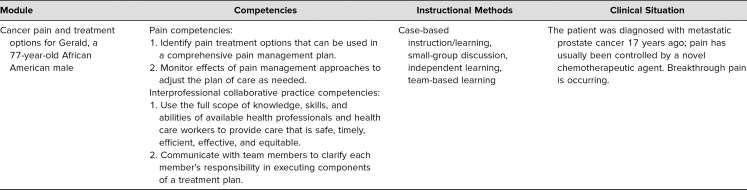
Case Description and Instructional Methods

### IPE

One of the goals of this module was to demonstrate the interprofessional team experience and to reflect on interprofessional collaborative practice. Many learners had already worked with other health care professionals in their clinical experiences had not been part of an interprofessional collaborative team. A frequent comment by students who participated in IPE and practice activities was “We don't really see this in ‘real practice.’” This activity was designed to allow students to work collaboratively on a pain-related case and to identify barriers to and facilitators of interprofessional collaborative practice.

### Implementation Strategies and Learner Levels

We recommend that this module be part of an interprofessional learning experience with teams of eight to 12 students from multiple professions (e.g., dentistry, medicine, nursing, pharmacy, social work) for small-group discussions. Group size may vary depending on the number of professions taking part, with the goal of including two representatives from each profession per team. Ideally, the learners will be at similar levels (e.g., second-year nurse practitioner students, third- and fourth-year medical students). However, since the experience does not target clinical expertise but rather competencies such as assessing patient preferences, integrating diverse perspectives into care planning, and working with an interprofessional team, there is flexibility on the level and type of learners chosen to participate.

### Facilitator Planning

If possible, this module should be facilitated by an interprofessional faculty team. However, the module can be led by a single facilitator, and facilitators do not need to have expertise in pain management. To prepare for the in-person session, facilitators should review all of the materials, including the independent learning module, facilitator guide, appendix items, and recommended resources. The independent learning module includes an overview of the topic of cancer pain for those without expertise in that area.

### Independent Learning Module

To optimize the learning experience utilizing the flipped classroom model, a 15-minute web-based presentation on cancer pain management and treatment (Appendix B) was included as a resource for learners to complete prior to the in-person session. This independent web-based module provided students with foundational knowledge tied to the learning objectives, group activities, and discussions. A brief quiz was included as an attachment in the facilitator guide (Appendix A, attachment D) to identify areas requiring additional discussion during the independent learning review session. We recommend that this anonymous quiz be administered through an online survey program of the facilitator's choice with the results sent directly to the facilitator prior to the in-person training. Facilitators may consider adapting the survey to include questions that assess students' perceptions of their strengths and weaknesses as these relate to pain, symptom management, and interdisciplinary care, as well as what students hope to gain by participating in the session. Other recommended handouts to consider distributing prior to the in-person session include the Brief Pain Inventory^16^ and the PHQ-9 Questionnaire for Depression Scoring and Interpretation Guide.^17^

### Activity Schedule

• Facilitators' presession planning (should occur at least 1 week prior to session):
  ○ Review facilitator's guide (Appendix A), independent learning activities and resources (Appendices B–G), and other resources: 30 minutes.
  ○ If multiple individuals are facilitating the session, meet as a group to review material and identify point person for each module activity: 45 minutes.
  ○ Send link to independent learning activities 1 week prior to session: 5 minutes.
  ○ Review independent learning activity quiz results: 10 minutes.
• Learners' presession activities:
  ○ Complete independent learning on cancer pain treatment and management (Appendix B).
  ○ Complete anonymous quiz (Appendix A, attachment D).
• In-person session (120 minutes):
  ○ Introduction for case and interprofessional icebreaker: 15 minutes.
  ○ Orientation for Gerald experience: competencies (Appendix D): 5 minutes.
  ○ Independent learning review: 10 minutes.
  ○ Quick reference—Gerald (Appendix E): 5 minutes.
  ○ 1–2–4 activity: 10 minutes.
  ○ Facilitator-led debrief: 15 minutes.
  ○ Student preparation of problem list and treatment plan: 15 minutes.
  ○ Groups present their treatment plans and discuss: 15 minutes.
  ○ Gerald—part II PowerPoint (Appendix F): 5 minutes.
  ○ Student preparation of treatment plan adjustment: 10 minutes.
  ○ Facilitator-led student discussion (Appendix F): 10 minutes.
  ○ Facilitator recap (Appendix F): 5 minutes.
• Postsession activity:
  ○ Session evaluation (Appendix G).

## Results

A mixed-methods approach was used to evaluate this module. An evaluation team from the UC Davis Clinical and Translational Science Center led the evaluation design and analysis. Data were collected via focus groups, direct observation, and a postsession survey completed by learners.

The cancer pain and treatment module was piloted with nine interprofessional learners: in the first pilot, two nurse practitioner or physician assistant students, three pharmacy students, two medical students, and two social work students. The second pilot also had nine learners with the same interprofessional breakdown as the first pilot. The Pain Knowledge and Beliefs Questionnaire^13^ was administered at the completion of each session. Using a 5-point Likert scale (1 = *strongly disagree,* 5 = *strongly agree*), the mean score on a set of questions about the value of the educational module was 4.4 for the July 2015 pilot and 4.6 for the January 2016 pilot. Please see Table 2 for full results. Additional comments collected from learners on the survey are included in Table 3.

**Table 2.**
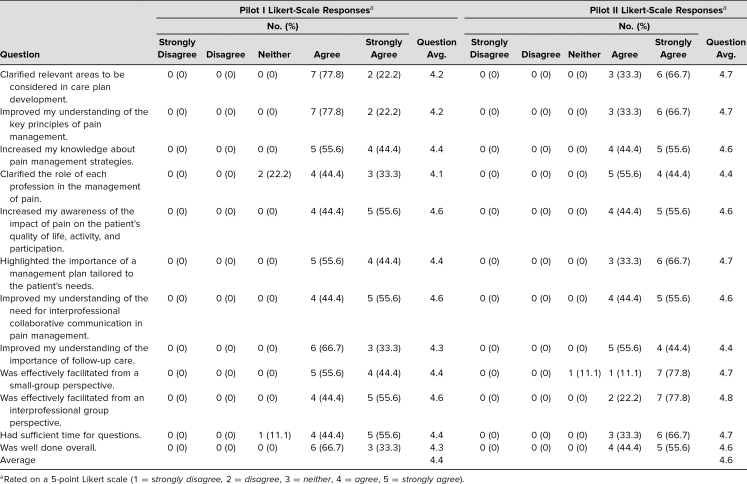
Pain Knowledge and Belief Questionnaire Results for Cancer Pain and Treatment Module: Pilots I and II

**Table 3.**
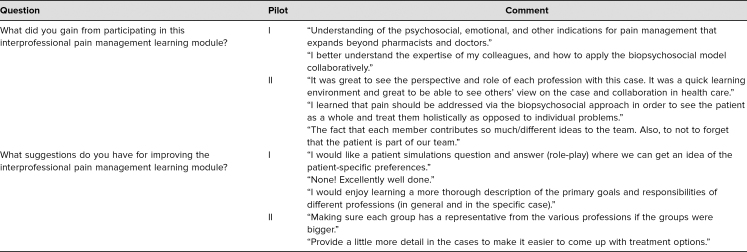
Sample Comments From Learners in Cancer Pain and Treatment Module Pilots I and II

## Discussion

This IPE module used a case study about cancer pain treatment to provide a framework in which students from differing health professions were able to learn with, from, and about each other. Survey responses from participants in the pilot programs of this module expressed clear satisfaction with the content and approach and noted increased knowledge about both IPE and treating cancer pain. Survey comments included the following:

• “The information regarding cancer pain and IPE was informative.”
• “The percentage of cancer patients who experience pain was higher than I expected.”
• “Depression and pain was a new concept for me.”
• “I learned about breakthrough pain and how to manage different types of pain.”

These pilots demonstrate that learning activities targeting two nationally recognized sets of core competencies—core competencies in pain management for prelicensure clinical education and core competencies for interprofessional practice*—*can be incorporated into an interprofessional learning experience.

### Limitations and Next Steps

Additional time for faculty planning and preparation may be required to cofacilitate IPE activities. It may be helpful to provide IPE faculty development workshops to support faculty commitment from different disciplines. Challenges in implementing the modules included scheduling learners within disparate professional training programs who were on differing schedules. Ensuring implementation also required that we have the support of leadership within the educational programs from each of the represented professions. Although we suspected it might be difficult for the different groups to feel comfortable together, it seemed that this was not the case after we had the participants introduce themselves and discuss what they hoped to learn from students in different professions.

Despite being unable to determine if the flipped classroom strategy led to higher scores in the second pilot, this format provided the learners with the advantage of some degree of prelearning that may have allowed for more time to have substantive discussions regarding the care plan and potential resources. Other limitations of this module included developing and piloting with a select group of health professions and levels. Additionally, no baseline assessment of student readiness for team-based learning or understanding of pain management was conducted prior to piloting the module. Although the session evaluation (Appendix G) included an evaluation of student improvement and performance in both team-based learning and pain treatment and management, the lack of a baseline resulted in an incomplete assessment of learners at the conclusion of the module and may have produced more subjective results than an exit survey paired with a premodule self-assessment. Moreover, we were unable to address outcome measures that assessed the achievement of ability of learners to construct a problem list and treatment plan for the management of pain, the ability to adjust a plan of care, or the ability to communicate effectively. Targeting meaningful outcomes such as these will enhance future revised versions of the module. Further improvements to the module could include piloting with additional professions, as well as developing a premodule self-assessment addressing both team-based learning and pain management knowledge.

## Appendices

Facilitator Guide.docxCancer Pain & Treatment Module folderModule Access Instructions.docxHandout I.docxHandout II.docxPresentation.pptxSession Evaluation.docx
All appendices are peer reviewed as integral parts of the Original Publication.
