# Supplementary material for: Cancer Pain Treatment and Management: An Interprofessional Learning Module for Prelicensure Health Professional Students
Source: MedEdPORTAL. 2020 Sep 9;16:10953. doi: 10.15766/mep_2374-8265.10953 (PMC7485910; doi:10.15766/mep_2374-8265.10953)
Supplement: Supplementary file 1 — Facilitator Guide.docxCancer Pain & Treatment Module folderModule Access Instructions.docxHandout I.docxHandout II.docxPresentation.pptxSession Evaluation.docx [file mep_2374-8265.10953-s001.zip › B. Cancer Pain & Treatment Module folder/Cancer Pain and Treatment Options - Presenter output/presentation.html]

Cancer Pain and Treatment Options
