# Supplementary material for: Cancer Pain Treatment and Management: An Interprofessional Learning Module for Prelicensure Health Professional Students
Source: MedEdPORTAL. 2020 Sep 9;16:10953. doi: 10.15766/mep_2374-8265.10953 (PMC7485910; doi:10.15766/mep_2374-8265.10953)
Supplement: Supplementary file 1 — Facilitator Guide.docxCancer Pain & Treatment Module folderModule Access Instructions.docxHandout I.docxHandout II.docxPresentation.pptxSession Evaluation.docx [file mep_2374-8265.10953-s001.zip › C. Module Access Instructions.docx]

Appendix C: Accessing the Independent Learning Module

**Preparing Learners for the Session: Independent Learning Module**

To optimize the learning experience, a 15-minute web-based presentation on ***Cancer Pain***

***and Treatment Options (Appendix B)*** is included

as a resource for learners to complete prior to the Additional Resources

- University of Washington IPE resources: [http://www.wish.washington.edu/services/ipe_faculty_res ources.](http://www.wish.washington.edu/services/ipe_faculty_resources)
- University of Texas IPE Competency Video Series: [https://www.youtube.com/channel/UCvpF6R6- q7wLenkqE8qWHLg](https://www.youtube.com/channel/UCvpF6R6-q7wLenkqE8qWHLg)
- Brief Pain Inventory
- (PHQ-9): Questionnaire for Depression Scoring and

Interpretation Guide

in-person session. This independent learning module provides learners with foundational knowledge that is tied to the group activities and discussions. A brief quiz is included (Facilitator Guide, Attachment D) to identify areas that may require additional discussion

during the “*Independent Learning Review*” session. It is recommended that this

anonymous quiz is administered through an online survey program of your choice with the results sent directly to the facilitator prior to the in-person training. Facilitators may consider requiring prelearning activities on other topics, such as interprofessional education (IPE), the brief pain inventory, and the PHQ-9. Select examples of additional resources are listed in the box above.

***To access Cancer Pain and Treatment Options:***

# Download Cancer Pain and Treatment Options.zip

If asked to "Open" or "Save" the file, select **Save**, then **Open Folder**. If not, continue to **Step 2**

1. Locate the downloaded .zip file (check your Downloads folder)
2. Extract all files from the downloaded .zip file ("unzip" the file) For PCs (Windows):

Right-click file and select **Extract All**, then follow the instructions

# OR

At the top of the File Explorer window under **Compressed Folder Tools** select

**Extract**, then **Extract All**

For Macs:

Double click the .zip file

1. From the extracted folder, open **presentation_html5.html** using a web browser (Google Chrome is recommended and Microsoft Edge does not work)
