# Supplementary material for: Cancer Pain Treatment and Management: An Interprofessional Learning Module for Prelicensure Health Professional Students
Source: MedEdPORTAL. 2020 Sep 9;16:10953. doi: 10.15766/mep_2374-8265.10953 (PMC7485910; doi:10.15766/mep_2374-8265.10953)
Supplement: Supplementary file 1 — Facilitator Guide.docxCancer Pain & Treatment Module folderModule Access Instructions.docxHandout I.docxHandout II.docxPresentation.pptxSession Evaluation.docx [file mep_2374-8265.10953-s001.zip › D. Handout I.docx]

# Handout I

**Interprofessional Pain Management Learning Module**

***Gerald Dubois***

# Pain Management Core Competencies1:

1. **Pain Competency 3.2:** Identify pain treatment options that can be used in a comprehensive pain management plan.
2. **Pain Competency 3.5:** Monitor effects of pain management approaches to adjust the plan of care as needed.

# Interprofessional Collaborative Practice Competencies (ICPC)2:

1. **Interprofessional Practice Competency RR5:** Use the full scope of knowledge, skills, and abilities of available health professionals and healthcare workers to provide care that is safe, timely, efficient, effective, and equitable.
2. **Interprofessional Practice Competency RR6:** Communicate with team members to clarify each member’s responsibility in executing components of a treatment plan.

RR=Roles and responsibilities

# Learning Objectives:

After completing this case-study learning experience, participants should be able to:

1. Construct a problem list and treatment plan for initial management of a complex pain case presentation.
2. Use the biopsychosocial model to create an effective patient-centered pain management plan.
3. In the context of an interprofessional team, adjust a plan of care in light of feedback gained during ongoing assessment of pain, function, and overall systems.
4. Recognize the benefits of patient-centered, team-based care.
5. Communicate with other health professionals in a responsive and responsible manner that supports a team approach to care.
6. Fishman SM, Young HM, Lucas Arwood E, et al. Core competencies for pain management: results of an interprofessional consensus summit. *Pain Med*. Jul 2013;14(7):971-981. <http://www.ncbi.nlm.nih.gov/pmc/articles/PMC3752937/>
7. Interprofessional Education Collaborative Expert Panel. (2011). Core competencies for interprofessional collaborative practice: Report of an expert panel. Washington, D.C.: Interprofessional Education

Collaborative. <https://ipecollaborative.org/uploads/IPEC-Core-Competencies.pdf>
