# Supplementary material for: Cancer Pain Treatment and Management: An Interprofessional Learning Module for Prelicensure Health Professional Students
Source: MedEdPORTAL. 2020 Sep 9;16:10953. doi: 10.15766/mep_2374-8265.10953 (PMC7485910; doi:10.15766/mep_2374-8265.10953)
Supplement: Supplementary file 1 — Facilitator Guide.docxCancer Pain & Treatment Module folderModule Access Instructions.docxHandout I.docxHandout II.docxPresentation.pptxSession Evaluation.docx [file mep_2374-8265.10953-s001.zip › E. Handout II.docx]

**Handout II – Quick Reference**

Name: Gerald (Gerry) Dubois

**Age:** 77-years old

**Gender:** Male

**Race/Ethnicity:** African American

**Marital Status**: Married

**Occupation:** Retired Large Animal Veterinarian

# Chief Complaint:

Breakthrough pain is occurring despite the Acetaminophen/Codeine he was prescribed, which has also resulted in constipation. He takes 1-2, two times per day.

# History of Presenting Illness:

Gerald was diagnosed with metastatic prostate cancer 17 years ago, but the cancer has been held in check by a novel chemotherapeutic agent. Now, however, he has severe axial lumbar breakthrough pain that, he is told, is due to osteoblastic lesions on his lower spine. He describes that he was an athlete in college and continued to be an active adult who enjoyed golf, long walks, and gardening. Because of the pain, he can’t play golf or do any of his former activities and is depressed about that, in addition to being tired from disrupted sleep. The constipation, he says, “just makes everything worse. I can’t go on like this.” His wife is with him and shares that he rarely goes out anymore because he “doesn’t want to be seen.” She is struggling with keeping up with what she needs to do and still care for her family.

| **Assessment/Vitals** | **Resources** |
| --- | --- |
| Pain score: 9/10 | Brief Pain Inventory (BPI)  Score range: 0 = No pain to 10 = “Pain as bad as you can imagine it”  *For information on the Brief Pain Inventory, please see: Tan, G., Jensen, M. P., Thornby, J. I., & Shanti, B. F. (2004). Validation of the Brief Pain Inventory for chronic nonmalignant pain. [Validation Studies]. J Pain, 5(2),*  *133-137.* |
| Depression screen score (PHQ-9): 19/27 | Patient Health Questionnaire (PHQ-9) Score range: 0 to 27  Score interpretation: Brief, self-administered questionnaire for screening, diagnosis, monitoring and measuring severity of depression in clinical practice.  0-4: Minimal  5-9: Mild  10-14: Moderate  15-19: Moderately severe  20-27: Severe |
| **Vitals** | **Normal Range** |
| Heart rate: 60 | 60 to 100 beats per minute |
| Blood pressure: 110/80 | 90/60 mm/Hg to 120/80 mm/Hg. Blood pressure is variable for individuals and age groups. It  may fluctuate and increase with exercise, illness, injury, pain, and emotions. |
| Temperature: 99.0 (F) / 37.2 (C) | 97.8 - 99.1 degrees Fahrenheit [36.6 – 37.3 degrees Celsius] |
| Oxygen saturation: 100% | 90-100% |
| Respiratory rate: 10 | 12-18 breaths per minute |
| **Past Medical History** |  |
| CAD with CABG 20 years ago; cardiac cath within last year shows no change in coronary artery patency; pulmonary embolism and deep vein thrombosis: 4 months ago, now medicated with Xarelto; hypertension; mild congestive heart failure  with paroxysmal atrial fibrillation. | CAD = [coronary artery disease.](http://www.medilexicon.com/medicaldictionary.php?s=coronary%2Bartery%2Bdisease) CABG = [coronary artery bypass graft](http://www.medilexicon.com/medicaldictionary.php?s=coronary%2Bartery%2Bbypass%2Bgraft) |
| **Medications** | **Medication Use** |
| Metoprolol Succinate – 25 mg, once daily | Beta blocker used to treat angina (chest pain) and hypertension (high blood pressure) |
| Carvedilol (Coreg) 6.25 mg BID | Beta blocker used to treat heart failure and hypertension (high blood pressure). |
| Lisinopril 10 mg daily | ACE inhibitor used to treat high blood pressure (hypertension), congestive heart failure, and to  improve survival after a heart attack. |
| Rivaroxaban (Xarelto) 20 mg daily | Anticoagulant used to prevent or treat a type of blood clot called deep vein thrombosis (DVT), which can lead to blood clots in the lungs (pulmonary embolism). Persons with  cancer have an increased risk for DVT. |
| Acetaminophen/Codeine (300/30); 1-2  every 4-6 hours as needed | Schedule II opioid and analgesic/antipyretic combination used to relieve mild to moderately  severe pain. |
