# Supplementary material for: Cancer Pain Treatment and Management: An Interprofessional Learning Module for Prelicensure Health Professional Students
Source: MedEdPORTAL. 2020 Sep 9;16:10953. doi: 10.15766/mep_2374-8265.10953 (PMC7485910; doi:10.15766/mep_2374-8265.10953)
Supplement: Supplementary file 1 — Facilitator Guide.docxCancer Pain & Treatment Module folderModule Access Instructions.docxHandout I.docxHandout II.docxPresentation.pptxSession Evaluation.docx [file mep_2374-8265.10953-s001.zip › G. Session Evaluation.docx]

**Session Evaluation**

**Thank you for completing this evaluation of the learning module. Your feedback will be used to guide revisions of the modules and overall program.**

**About You** (circle response that applies):

**Profession**: NP Pharmacy Student

Medical Student

Social Work Student

1. Please indicate how much you agree with the following statements by circling your response using the scale provided, where **1 = strongly disagree** and **5 = strongly agree**.

|  | Strongly Disagree | Disagree | Neither Agree or  Disagree | Agree | Strongly Agree |
| --- | --- | --- | --- | --- | --- |
| a. Clarified relevant areas to be considered in care plan development | 1 | 2 | 3 | 4 | 5 |
| b. Improved my understanding of the key principles of pain management | 1 | 2 | 3 | 4 | 5 |
| c. Increased my knowledge about pain management strategies | 1 | 2 | 3 | 4 | 5 |
| d. Clarified the role of each profession in the management of pain | 1 | 2 | 3 | 4 | 5 |
| e. Increased my awareness of the impact of pain on the patient’s quality of life, activity and participation | 1 | 2 | 3 | 4 | 5 |
| f. Highlighted the importance of a management plan tailored to the patient’s need | 1 | 2 | 3 | 4 | 5 |
| g. Improved my understanding of the need for interprofessional collaborative communication in pain management | 1 | 2 | 3 | 4 | 5 |
| h. Improved my understanding of the importance of follow-up care | 1 | 2 | 3 | 4 | 5 |
| i. Was effectively facilitated from a ‘‘small group” perspective | 1 | 2 | 3 | 4 | 5 |
| j. Was effectively facilitated from an ‘‘interprofessional group” perspective | 1 | 2 | 3 | 4 | 5 |
| k. Had sufficient time for questions | 1 | 2 | 3 | 4 | 5 |
| l. Was overall well done | 1 | 2 | 3 | 4 | 5 |

1. What did you gain from participating in this interprofessional pain management learning module?
2. What suggestions do you have for improving the interprofessional pain management learning module?
