# Supplementary figures and images for: Cancer Pain Treatment and Management: An Interprofessional Learning Module for Prelicensure Health Professional Students
Source: MedEdPORTAL. 2020 Sep 9;16:10953. doi: 10.15766/mep_2374-8265.10953 (PMC7485910; doi:10.15766/mep_2374-8265.10953)

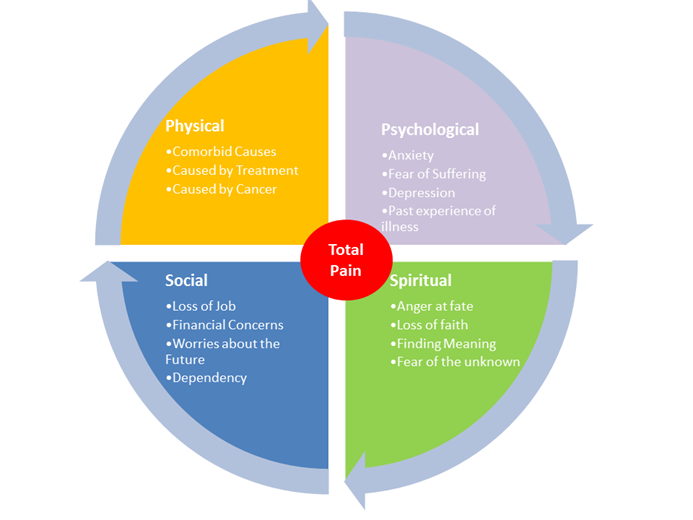

Supplement: Supplementary file 1 — Facilitator Guide.docxCancer Pain & Treatment Module folderModule Access Instructions.docxHandout I.docxHandout II.docxPresentation.pptxSession Evaluation.docx [file mep_2374-8265.10953-s001.zip › B. Cancer Pain & Treatment Module folder/Cancer Pain and Treatment Options - Presenter output/mobile/5jwYeWXiw9G_80_DX1386_DY1386_CX693_CY520.png]

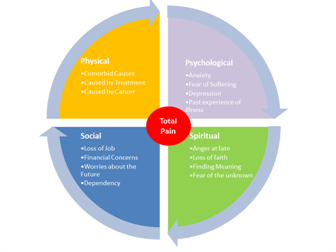

Supplement: Supplementary file 1 — Facilitator Guide.docxCancer Pain & Treatment Module folderModule Access Instructions.docxHandout I.docxHandout II.docxPresentation.pptxSession Evaluation.docx [file mep_2374-8265.10953-s001.zip › B. Cancer Pain & Treatment Module folder/Cancer Pain and Treatment Options - Presenter output/mobile/5jwYeWXiw9G_None_336252_80_DX672_DY672_CX336_CY252.png]

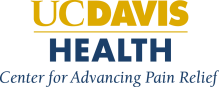

Supplement: Supplementary file 1 — Facilitator Guide.docxCancer Pain & Treatment Module folderModule Access Instructions.docxHandout I.docxHandout II.docxPresentation.pptxSession Evaluation.docx [file mep_2374-8265.10953-s001.zip › B. Cancer Pain & Treatment Module folder/Cancer Pain and Treatment Options - Presenter output/mobile/67eHfT4spQi.png]

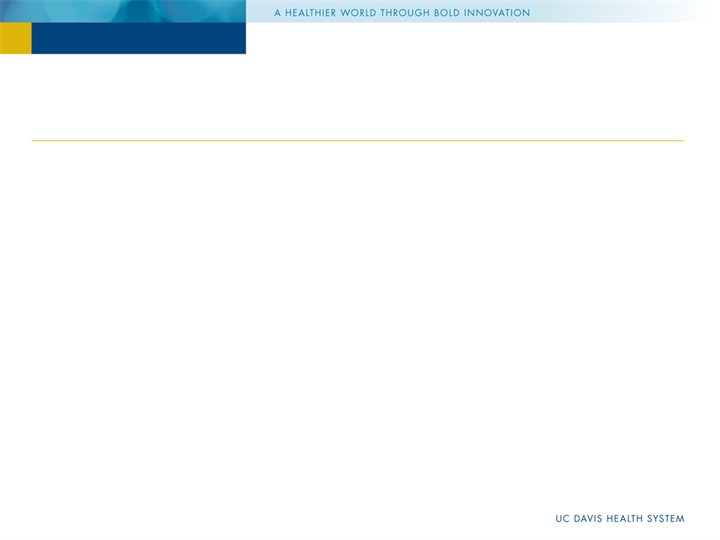

Supplement: Supplementary file 1 — Facilitator Guide.docxCancer Pain & Treatment Module folderModule Access Instructions.docxHandout I.docxHandout II.docxPresentation.pptxSession Evaluation.docx [file mep_2374-8265.10953-s001.zip › B. Cancer Pain & Treatment Module folder/Cancer Pain and Treatment Options - Presenter output/mobile/6IDV1IEtWwC_5dxUExJ3OTe_80_DX1440_DY1440_CX720_CY540.png]

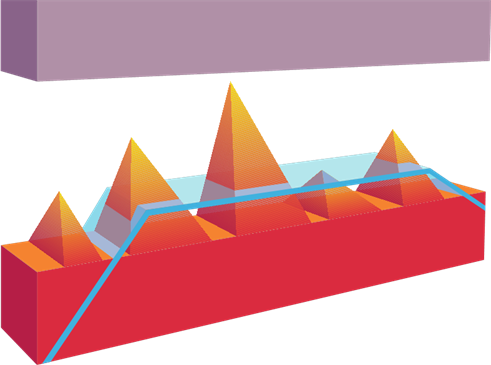

Supplement: Supplementary file 1 — Facilitator Guide.docxCancer Pain & Treatment Module folderModule Access Instructions.docxHandout I.docxHandout II.docxPresentation.pptxSession Evaluation.docx [file mep_2374-8265.10953-s001.zip › B. Cancer Pain & Treatment Module folder/Cancer Pain and Treatment Options - Presenter output/mobile/6IDV1IEtWwC_5fnlUG0ShH9_80_DX982_DY982_CX491_CY365.png]

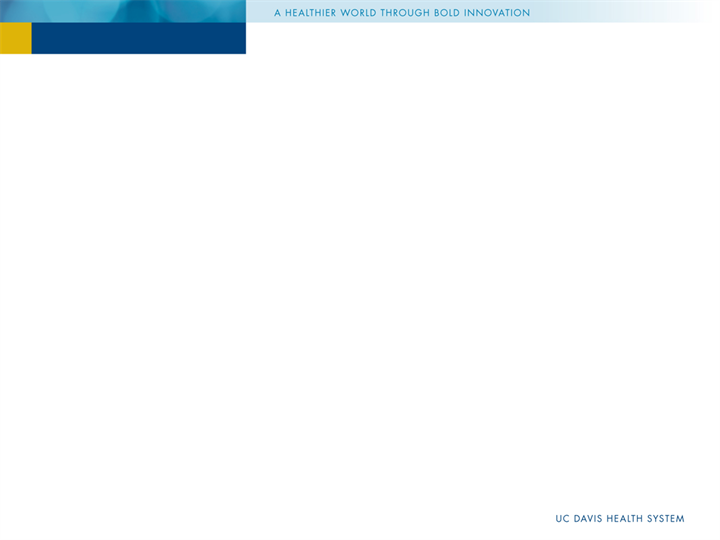

Supplement: Supplementary file 1 — Facilitator Guide.docxCancer Pain & Treatment Module folderModule Access Instructions.docxHandout I.docxHandout II.docxPresentation.pptxSession Evaluation.docx [file mep_2374-8265.10953-s001.zip › B. Cancer Pain & Treatment Module folder/Cancer Pain and Treatment Options - Presenter output/mobile/6IDV1IEtWwC_5tjRNe8KdbA_80_DX1440_DY1440_CX720_CY540.png]

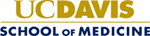

Supplement: Supplementary file 1 — Facilitator Guide.docxCancer Pain & Treatment Module folderModule Access Instructions.docxHandout I.docxHandout II.docxPresentation.pptxSession Evaluation.docx [file mep_2374-8265.10953-s001.zip › B. Cancer Pain & Treatment Module folder/Cancer Pain and Treatment Options - Presenter output/mobile/6IDV1IEtWwC_5VXEXpyJXi5_80_DX300_DY300_CX150_CY36.png]

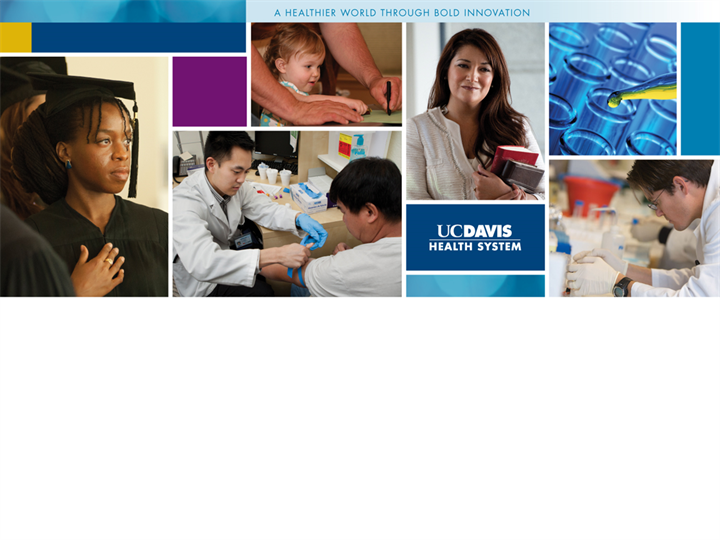

Supplement: Supplementary file 1 — Facilitator Guide.docxCancer Pain & Treatment Module folderModule Access Instructions.docxHandout I.docxHandout II.docxPresentation.pptxSession Evaluation.docx [file mep_2374-8265.10953-s001.zip › B. Cancer Pain & Treatment Module folder/Cancer Pain and Treatment Options - Presenter output/mobile/6IDV1IEtWwC_5WHr7H9ZgWu_80_DX1440_DY1440_CX720_CY540.png]

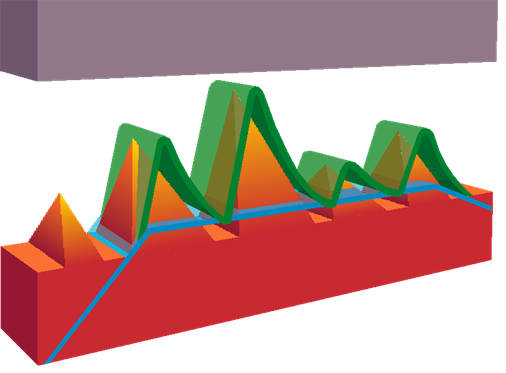

Supplement: Supplementary file 1 — Facilitator Guide.docxCancer Pain & Treatment Module folderModule Access Instructions.docxHandout I.docxHandout II.docxPresentation.pptxSession Evaluation.docx [file mep_2374-8265.10953-s001.zip › B. Cancer Pain & Treatment Module folder/Cancer Pain and Treatment Options - Presenter output/mobile/6IDV1IEtWwC_63JerStY2uJ_80_DX1012_DY1012_CX506_CY373.png]

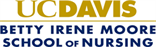

Supplement: Supplementary file 1 — Facilitator Guide.docxCancer Pain & Treatment Module folderModule Access Instructions.docxHandout I.docxHandout II.docxPresentation.pptxSession Evaluation.docx [file mep_2374-8265.10953-s001.zip › B. Cancer Pain & Treatment Module folder/Cancer Pain and Treatment Options - Presenter output/mobile/6IDV1IEtWwC_6EUefG78vTk_80_DX312_DY312_CX156_CY47.png]

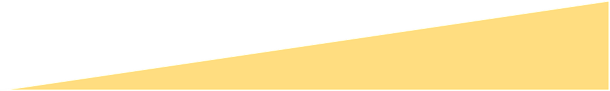

Supplement: Supplementary file 1 — Facilitator Guide.docxCancer Pain & Treatment Module folderModule Access Instructions.docxHandout I.docxHandout II.docxPresentation.pptxSession Evaluation.docx [file mep_2374-8265.10953-s001.zip › B. Cancer Pain & Treatment Module folder/Cancer Pain and Treatment Options - Presenter output/mobile/6IDV1IEtWwC_sl263shp180227_80_DX1220_DY1220_CX610_CY91.png]

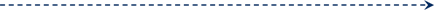

Supplement: Supplementary file 1 — Facilitator Guide.docxCancer Pain & Treatment Module folderModule Access Instructions.docxHandout I.docxHandout II.docxPresentation.pptxSession Evaluation.docx [file mep_2374-8265.10953-s001.zip › B. Cancer Pain & Treatment Module folder/Cancer Pain and Treatment Options - Presenter output/mobile/6IDV1IEtWwC_sl263shp180241_80_DX868_DY868_CX434_CY10.png]

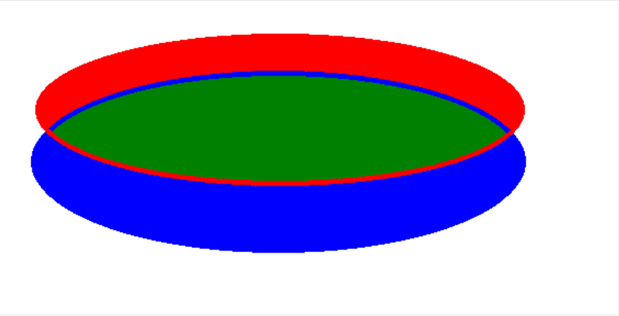

Supplement: Supplementary file 1 — Facilitator Guide.docxCancer Pain & Treatment Module folderModule Access Instructions.docxHandout I.docxHandout II.docxPresentation.pptxSession Evaluation.docx [file mep_2374-8265.10953-s001.zip › B. Cancer Pain & Treatment Module folder/Cancer Pain and Treatment Options - Presenter output/mobile/6IDV1IEtWwC_sl270shp37892_80_DX1238_DY1238_CX619_CY316.png]

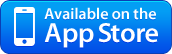

Supplement: Supplementary file 1 — Facilitator Guide.docxCancer Pain & Treatment Module folderModule Access Instructions.docxHandout I.docxHandout II.docxPresentation.pptxSession Evaluation.docx [file mep_2374-8265.10953-s001.zip › B. Cancer Pain & Treatment Module folder/Cancer Pain and Treatment Options - Presenter output/mobile/app_store.png]

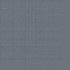

Supplement: Supplementary file 1 — Facilitator Guide.docxCancer Pain & Treatment Module folderModule Access Instructions.docxHandout I.docxHandout II.docxPresentation.pptxSession Evaluation.docx [file mep_2374-8265.10953-s001.zip › B. Cancer Pain & Treatment Module folder/Cancer Pain and Treatment Options - Presenter output/mobile/linen_background.jpg]

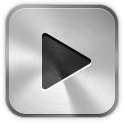

Supplement: Supplementary file 1 — Facilitator Guide.docxCancer Pain & Treatment Module folderModule Access Instructions.docxHandout I.docxHandout II.docxPresentation.pptxSession Evaluation.docx [file mep_2374-8265.10953-s001.zip › B. Cancer Pain & Treatment Module folder/Cancer Pain and Treatment Options - Presenter output/mobile/mobile_icon.png]

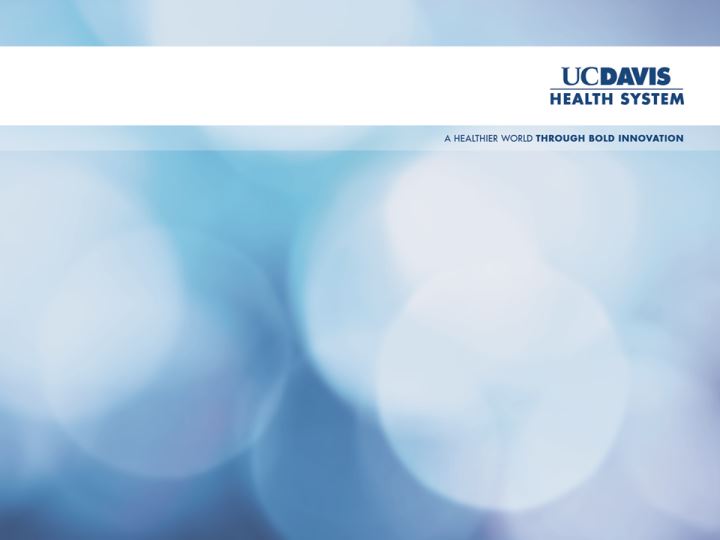

Supplement: Supplementary file 1 — Facilitator Guide.docxCancer Pain & Treatment Module folderModule Access Instructions.docxHandout I.docxHandout II.docxPresentation.pptxSession Evaluation.docx [file mep_2374-8265.10953-s001.zip › B. Cancer Pain & Treatment Module folder/Cancer Pain and Treatment Options - Presenter output/mobile/Slide6IDV1IEtWwC_d1cl1MailTitleSlide.jpg]

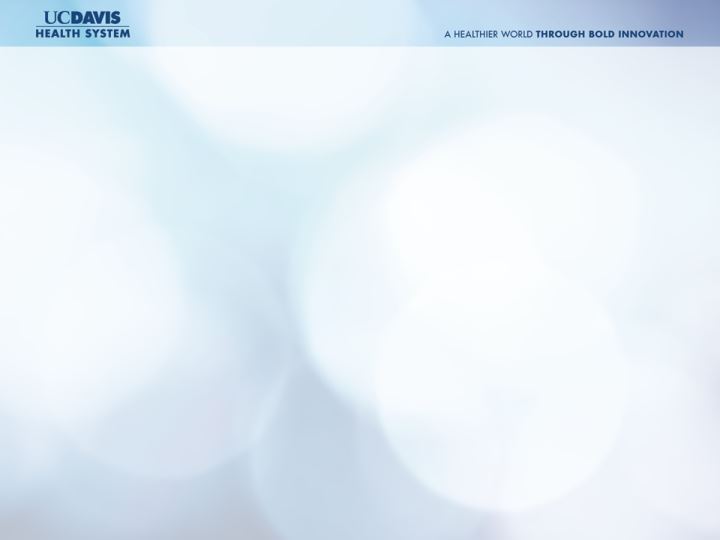

Supplement: Supplementary file 1 — Facilitator Guide.docxCancer Pain & Treatment Module folderModule Access Instructions.docxHandout I.docxHandout II.docxPresentation.pptxSession Evaluation.docx [file mep_2374-8265.10953-s001.zip › B. Cancer Pain & Treatment Module folder/Cancer Pain and Treatment Options - Presenter output/mobile/Slide6IDV1IEtWwC_d1cl5Blank.jpg]

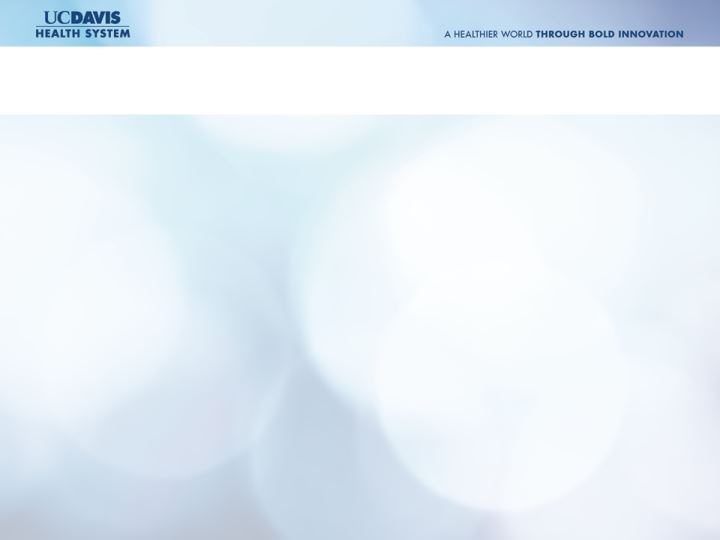

Supplement: Supplementary file 1 — Facilitator Guide.docxCancer Pain & Treatment Module folderModule Access Instructions.docxHandout I.docxHandout II.docxPresentation.pptxSession Evaluation.docx [file mep_2374-8265.10953-s001.zip › B. Cancer Pain & Treatment Module folder/Cancer Pain and Treatment Options - Presenter output/mobile/Slide6IDV1IEtWwC_d1sm.jpg]

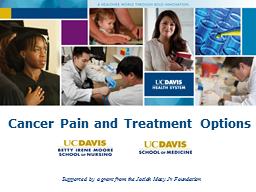

Supplement: Supplementary file 1 — Facilitator Guide.docxCancer Pain & Treatment Module folderModule Access Instructions.docxHandout I.docxHandout II.docxPresentation.pptxSession Evaluation.docx [file mep_2374-8265.10953-s001.zip › B. Cancer Pain & Treatment Module folder/Cancer Pain and Treatment Options - Presenter output/presentation_content/thumbnail.jpg]
